# OUT to IN: a body-oriented intervention program to promote preschoolers’ self-regulation and relationship skills in the outdoors

**DOI:** 10.3389/fpsyg.2023.1195305

**Published:** 2023-08-03

**Authors:** Guida Veiga, Daniela Guerreiro, José Marmeleira, Graça Duarte Santos, Clarinda Pomar

**Affiliations:** ^1^Departamento de Desporto e Saúde, Escola de Saúde e Desenvolvimento Humano, Universidade de Évora, Évora, Portugal; ^2^Comprehensive Health Research Centre (CHRC), Universidade de Évora, Évora, Portugal; ^3^Departamento de Pedagogia e Educação, Escola de Ciências Sociais, Universidade de Évora, Évora, Portugal; ^4^Centro de Investigação em Educação e Psicologia, Universidade de Évora, Évora, Portugal

**Keywords:** social–emotional competence, psychomotricity, physical play, relaxation, mind–body, early childhood education

## Abstract

**Introduction:**

Time for movement and outdoor experiences has decreased in children’s daily lives. Nevertheless, a growing body of research has shown that body-oriented interventions and outdoor time benefit preschoolers’ social–emotional development, a foundation for mental health. OUT to IN is a body-oriented intervention program implemented outdoors, designed to promote preschoolers’ social–emotional competence. This study aimed to evaluate the effects of OUT to IN on preschoolers’ self-regulation and relationship skills.

**Methods:**

A cluster randomized trial with multi-method and multi-informant assessment was implemented including 233 children between 3 and 6 years (122 boys, *M*_age_ = 5.07 years), from 4 preschools (8 groups with OUT to IN intervention, 4 groups without intervention – control group). The 153 children allocated to the OUT to IN group participated in biweekly sessions for 10 weeks. OUT to IN sessions followed a body-oriented approach comprising exercise play, relaxation, and symbolization activities, implemented outdoors by a psychomotor therapist and the preschool teacher. Sessions enabled children to feel, observe and control their bodily states and understand the relationship between their bodies and emotions. Teachers participated in a brief course and on 20 biweekly relaxation sessions. Children’s self-regulation was measured through specific tasks and a parent questionnaire. Relationship skills (i.e., empathy, communication, cooperation and sociability) were measured through parents’ and preschool teachers’ questionnaires. Mann–Whitney test was used to study differences at baseline between the OUT to IN group and the control group, and to study differences in the 10-week changes between both groups. Wilcoxon Test was used for intragroup comparisons.

**Results:**

After the 10-week intervention period, children who participated in OUT to IN showed significant improvements on self-regulation and relationship skills (empathy, cooperation and sociability), in comparison to the control group who did not show any significant improvements. Large size effects (*η*^2^ > 0.14) were found for most of the variables related to self-regulation and small (*η*^2^ > 0.01), medium (*η*^2^ > 0.06) and large size effects (*η*^2^ > 0.14) were found for the variables related to relationship skills.

**Conclusion:**

OUT to IN showed to be an effective body-oriented intervention program in improving children’s self-regulation and relationship skills, which are recognized foundations for mental health and well-being.

## 1. Introduction

The early years are a prime time for social–emotional development. Throughout the preschool years, children improve their social–emotional competencies, such as empathizing with others’ feelings, adapting behaviors, thoughts, and emotions, communicating their emotions and mental states, and cooperating and socializing with peers. Social–emotional competence has critical implications for children’s adjustment and success (Shonkoff and Phillips, 2000), such that it is widely recognized as a central goal of preschool education and a target of several intervention programs.

Although most intervention programs have been implemented indoors, the potential of outdoor learning dates to Aristotle and Plato. Indeed, the importance of the outdoors for children’s development is highlighted by the term kindergarten (or garden of children) and is central to the educational theories of recognized pedagogues such as Rousseau, Froebel, or Pestalozzi (Allen, 2017). The outdoors is a highly stimulating setting where emotions can run high, and children feel freer to move, experiment and modulate the environment and their behavior (Veiga et al., 2020). A recent systematic review showed that natural environments facilitate children’s positive relationships and socially adaptive behaviors (Mygind et al., 2021). Moreover, contact with natural elements has been established to have a calming effect, reducing stress and improving mental health (Tillmann et al., 2018). Specifically, concerning outdoor-friendly early childhood environments, the systematic review of Johnstone et al. (2022) showed the benefits for children’s social–emotional competence, particularly self-regulation and relationship skills.

With so few years under their belts, preschoolers are still learning to inhabit their bodies. Actually, it is by engaging their bodies in doing, moving, acting, and interacting that children acquire knowledge and master their competencies. Indeed, play, dance, relaxation, and other body-oriented approaches have been increasingly implemented in early childhood education and are known to effectively promote preschoolers’ social–emotional competence (Dias Rodrigues et al., 2022a,b). Body-oriented interventions are supported by the intrinsic relationship between motion and emotion, integrating both sides in a balance of sensing and moving. As bodily sensations, bodily postures, gestures, and expressions are inherent components of the emotional experience, accessing such somatic information is critical to one’s identity and social–emotional development (Fuchs and Koch, 2014). Nonetheless, a recent systematic review (Dias Rodrigues et al., 2022b) focused on the effects of body-oriented interventions implemented in early-childhood education settings, showed inconsistent findings regarding the benefits of these interventions on self-regulation. Such inconsistency was related to differences in the dosage of the interventions, suggesting that a higher frequency is needed to improve self-regulation. Concerning relationship skills, there is moderate evidence for the improvements of body-oriented interventions on preschoolers’ empathy and social interaction, and limited evidence regarding the improvements in social cooperation (Dias Rodrigues et al., 2022b).

The most used body-oriented approach to improve preschoolers’ social–emotional competence is play (Dias Rodrigues et al., 2022a,b). Regarding play-based interventions, research reveals moderate evidence for improvements in empathy and social interaction, limited evidence for increased social cooperation and contradictory evidence for self-regulation (Dias Rodrigues et al., 2022a). It has been speculated that the type of play facilitated by the intervention might have a critical role and that physical play (particularly, exercise play) might be particularly beneficial concerning self-regulation (Dias Rodrigues et al., 2022a). Indeed, physical play has been argued to give preschoolers an important opportunity to feel their own bodily states, which are a central part of the emotional experience. Such opportunity is crucial for children to become aware of the relationship between their own bodily states and emotions, therefore promoting self-awareness and regulation (Veiga et al., 2022). Moreover, research has shown that exercise play, mainly when engaged with peers, is related to emotion understanding, emotion regulation and social competence (Lindsey and Colwell, 2013; Veiga et al., 2017).

Interventions based on relaxation have been also implemented in preschools. Contrary to play interventions, relaxation involves a more pronounced interoceptive approach, with a specific focus on body awareness and regulation which paves the way to self-awareness and regulation (Veiga, 2022). Despite the recent popularity of mindfulness (Flook et al., 2015), other approaches have also been implemented in preschools, such as breathing exercises or progressive muscle relaxation (Murray et al., 2018). However, studies focused on this type of body-oriented intervention are still scarce and with low methodological quality (Dias Rodrigues et al., 2022a). Although a recent systematic review showed moderate evidence that relaxation intervention does not improve self-regulation, the authors hypothesized that such lack of positive outcomes could be related to the short duration of the sessions (11–25 min) (Dias Rodrigues et al., 2022a). No studies have yet examined the effects of relaxation on preschoolers’ relationship skills.

Body-oriented interventions combining play and relaxation are scarce. To the best of our knowledge only one study has combined both approaches, however the intervention involved an externally-oriented type of play (i.e., loose parts play) and a relaxation moment, which was implemented indoors. Besides, the intervention had a short duration (5-day), and the effects on self-regulation and relationship skills were not examined (Lee et al., 2020).

Acknowledging the important role of exercise play and relaxation for social–emotional development, OUT to IN, a body-oriented intervention program, combining both approaches was designed to improve preschoolers’ social–emotional competence. OUT to IN is based on embodiment theory (e.g., Fuchs and Koch, 2014), aiming to help preschoolers enter a sensing, reflective and affective mode. Hence, bodily activities, i.e., exercise play and relaxation, are structured and facilitated in order to give children opportunities to integrate bodily (interoceptive and proprioceptive) feedback, to reflect on it, and express these corporeal experiences, verbally and non-verbally. Between activities, children are asked to sense their own body, and become aware of their bodily states (e.g., heart rate, temperature, muscle tone, breathing). Considering the above-mentioned potential of the outdoors for social–emotional well-being, the program was designed to be implemented outdoors.

The main purpose of this study was to examine the effects of OUT to IN on preschoolers’ self-regulation and relationship skills. More specifically, it was hypothesized that self-regulation, empathy, cooperation, sociability, and communication would benefit from a 10-week (20 sessions) body-oriented intervention which combines exercise play, relaxation, and symbolization activities.

## 2. Methods

### 2.1. Ethics statement

The study was approved by the research ethics board at the University of Évora (#20088), Portugal, and was carried out under the standards set by the Declaration of Helsinki (General Assembly of the World Medical Association, 2014). Written informed consent was provided by preschool teachers and parents for their participating children. Children gave their verbal consent. The collected data was fully encrypted to ensure the privacy of the participants.

### 2.2. Procedures

The directors of four preschool Portuguese institutions were asked for permission to conduct the current study at their school. After preschool teachers’ consent regarding their willingness to participate in the study, they presented the project to the parents and handed out the consent forms and the questionnaires. After parents gave their written consent, testing sessions with children were scheduled.

### 2.3. Study design

The study was registered at clinicaltrials.gov (#200088). A randomized trial with multi-method (tasks and questionnaires) and multi-informant (children, preschool teachers, parents) assessment was implemented to evaluate the effects of OUT to IN on preschoolers’ social–emotional competencies. The study was implemented in 4 Portuguese preschools in the second and third trimester (January–June) of 2020/2021 and 2021/22 preschool year, nested within 12 groups. The inclusion criteria were: (a) age between 3 and 6 years, (b) not have participated in a similar intervention program within the last 6 months, and (c) not have a condition that can affect the participation in the study.

Eight groups were randomly allocated to the intervention and 4 groups to the control group. Each preschool had, at least, an intervention group and a control group. Children allocated to the intervention group participated in biweekly sessions for 10 weeks (total = 20 sessions) and children were allocated to the control groups maintained their usual routine. After the end of the study, children from the control group participated in OUT to IN sessions.

### 2.4. Participants

**Figure 1:**
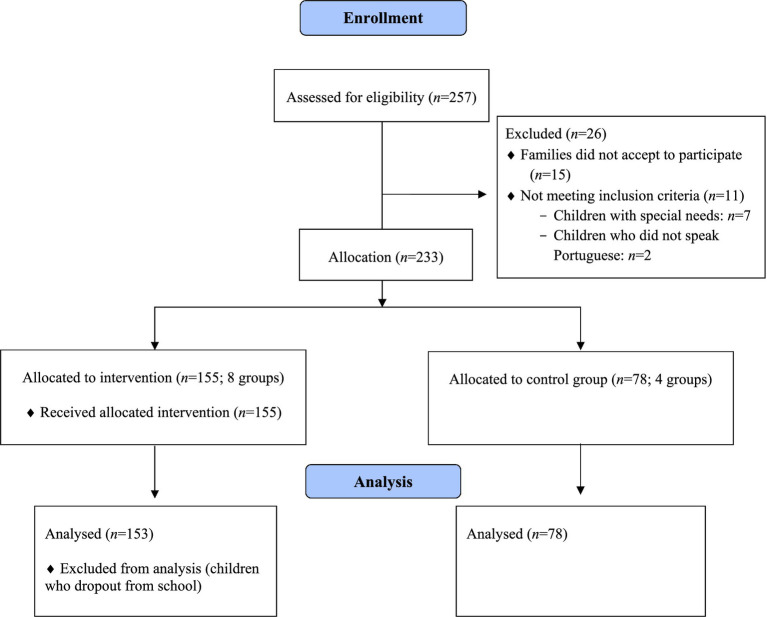
Flow diagram of recruitment and participation.

From a total of 257 families approached to participate in the study, only nine children did not meet the inclusion criteria (i.e., children with special needs, *n* = 7; refugee children who did not speak Portuguese, *n* = 2). Although these children participated in the intervention, they were not included for statistical analysis. As represented in Figure 1, two children dropped out school throughout the intervention period and 15 families did not accept to participate in the study, representing an acceptance rate of 92.2%. Regarding the remaining 233 children (122 boys; M_age_ = 5.07 years), the majority were Portuguese (*n* = 227). The nationality of the other children was other European (*n* = 1), Asiatic (*n* = 2), and South American (*n* = 3). The predominant level of maternal education was higher education (40.3%), the predominant level of paternal education was secondary education (44.6%), most children had brothers or sisters (74.5%) and lived in an urban area (79.7%).

Children were randomly allocated to the OUT to IN Group (*n* = 155; 8 groups with intervention) and to the Control Group (*n* = 78; 4 groups without intervention – control group). As represented in Table 1 no significant differences were found between groups, regarding age, gender, and sociodemographic measures. Also, as shown in Table 2 no differences were found between groups in terms of their self-regulation and relationship skills.

**Table 1:** Demographic characteristics of participants.

	Participants	OUT to IN group	Control group
	(*n* = 233)	(*n* = 153)	(*n* = 78)
Years of age (*M*, SD)	5.07 (0.84)	5.07 (0.82)	5.08 (0.87)
Years of age (range)	3.19–6.30	3.19–6.30	3.19–6.30
Boys (*n*)	122	80	42
Girls (*n*)	111	73	36
Maternal education (%)			
Basic education	11.7	12.6	9.9
Secondary education	36.4	34.8	39.4
Higher education	40.3	40.0	40.8
Master or Doctoral degree	11.7	12.6	9.9
Paternal education (%)			
Basic education	27.0	27.6	25.7
Secondary education	44.6	44.8	44.3
Higher education	21.6	21.6	21.4
Master or Doctoral degree	6.9	6.0	8.6

**Table 2:** Scores (baseline- and post- intervention), changes of scores, and effect sizes on self-regulation.

	Baseline (*M*, SD)		Post-intervention (10 weeks) (*M*, SD)		Difference between means, *M* (%95 CI)		Value of *p*
	OUT to IN group	Control group	OUT to IN group	Control group	OUT to IN group	Control group	
Children							
DN Task	0.61 (0.37)	0.57 (0.37)	0.91 (0.14)***	0.61 (0.34)	0.30 (−0.12; 1.00)	0.03 (−28.00, 29.00)	<0.001
HTKS Task	12.71 (16.64)	11.51 (15.71)	33.75 (16.98)***	13.07 (16.21)	21.02 (−16.00, 60.00)	1.55 (−28.00, 29.00)	<0.001
Parents							
Externalizing behaviors	1.62 (0.33)	1.65 (0.34)	1.56 (0.33)	1.61 (0.31)	−0.07 (−0.70, 1.30)	−0.04 (−1.00, 0.30)	0.120

^a^Inter-group comparisons at baseline through Mann–Whitney Test. *Intra-group comparisons through Wilcoxon Test (*p < 0.05; **p < 0.01; ***p < 0.001). *p* for 10- week changes between both groups through Mann–Whitney Test.

### 2.5. Procedures and materials

Children were tested individually in a quiet room of the school. Testing sessions took approximately 25 minutes and were video recorded. Children’s self-regulation was measured individually through specific tasks that are presented as games and a parent questionnaire. Relationship skills (i.e., empathy, cooperation, sociability and communication) were measured through parents’ and preschool teachers’ questionnaires. Due to sickness or holidays, some children failed the testing sessions. Also, some parents did not retrieve the questionnaires. As Little’s MCAR test (*p* > 0.05) indicated these missing values were random, all participants were included and listwise deletion was used for the cases with missing values.

#### 2.5.1. Self-regulation

Self-regulation was measured through the Day and Night task (DN; Gerstadt et al., 1994), the Head Toes Knees and Shoulders task (HTKS; McClelland et al., 2014) and the composite scale Externalizing Behaviors from the Strengths and Difficulties Questionnaire (SDQ; Goodman, 1997).

The DN is a simplified version of the Stroop test (Stroop, 1935) for younger children. It is a task that requires inhibitory control, involving the ability to inhibit a natural tendency to give a verbal response in accordance with the visual stimulus, but instead give a verbal response which is opposite to the visual command. Specifically, whenever the child sees a black card, with a moon and stars, he/she must say “day,” whenever the child sees a white card, with a brightly sun, must say “night.” The number of “day” and “night” cards is equal, and the cards are presented in an aleatory order. One point is assigned to each correct answer, totaling a maximum of 16 points. A higher classification reflects a better inhibitory control.

The HTKS task (McClelland et al., 2014) involves inhibitory control, working memory, and attention. The performance of the HTKS task requires the ability to inhibit the natural tendency to give a motor response in accordance with the verbal command, and to perform a motor response, which is opposite to the verbal command. For example, when the child is asked to touch the feet, he/she must touch the head and vice versa, when the child is asked to touch the shoulders, he/she must touch the knees and vice versa. The task involves three parts, each with 10 commands (feet/head; shoulders/knees; feet/head/shoulders/knees). Two points are assigned to each command correctly performed and one is assigned when the child can self-correct the performance, totaling a maximum of 60 points. A higher classification reflects a better inhibitory control.

Externalizing behaviors were obtained through a composite scale which comprises two scales of the Strengths and Difficulties Questionnaire (Goodman, 1997; Cronbach’ s alpha = 0.75): behavior problems (5 items; e.g., “Often has temper tantrums or hot tempers”; “Often fights with other children or bullies them”) and hyperactivity (5 items; e.g., Restless, overactive, cannot stay still for long; Constantly fidgeting or squirming). Parents were asked to rate their children’s behavior in a 3-point Likert scale, from 1 (not true) to 3 (certainly true). An average score of the 10 items is obtained and higher scores indicate poorer self-regulation.

#### 2.5.2. Relationship skills

The Empathy, Cooperation and Sociability were measured through the parents’ and preschool teachers’ Study on Social and Emotional Skills Questionnaire (SSES; Kankaraš and Suarez-Alvarez, 2019), a parent and preschool teacher report questionnaire. Parents and preschool teachers were asked to fill in the Empathy (e.g., “He/she can feel how others are feeling”; “He/she understands what others want”; Cronbach’ s alpha = 0.81, 0.85), Cooperation (e.g., He/she works well with others; he/she likes to help others; Cronbach’ s alpha = 0.62, 0.62), and Sociability subscales (e.g., He/she has many friends; He/she makes friends easily; Cronbach’ s alpha = 0.72, 0.49) in a 5-point Likert scale from one (completely disagree) to five (completely agree). Each scale is obtained by the average of the respective items. Higher scores indicate better relationship skills.

Emotion communication was obtained through the Emotion Vocabulary Questionnaire (EVQ; Ketelaar et al., 2015), a parent-report which assesses whether children know and use emotion and/or mental state words, in a 2-point Likert scale from 1 (no) to 2 (yes). The EVQ includes either basis emotions (e.g., happy, angry), complex emotions (e.g., jealous, disappointed), and mental state words (e.g., thinking, dreaming). A mean score across the items is calculated to indicate children’s emotion communication (Cronbach’ s alpha = 0.72).

#### 2.5.3. The OUT to IN program

OUT to IN comprises 20 body-oriented biweekly 40-min sessions facilitated by a psychomotor therapist in a pedagogical partnership with the preschool teacher. Sessions are implemented outdoors. All sessions are framed in a context of freedom, and self and mutual bodily resonance, according to the following structure: First, children are invited to engage in exercise play activities such as running, jumping, rolling, which are presented in a semi-directed approach, seeking to promote the sensing, exploration, and awareness of different bodily, rhythmic, and expressive movements. Second, children engage in ludic relaxation proposals such as stretching, observing, and controlling their own breathing, changing the levels of tension in different body segments, active-passive movements, etc., which help children to focus their attention on their body, sensing it and progressively learning to control it. Finally, children are asked to reflect on their bodily experiences and express themselves through different expressive mediators such as voice, movement, painting, or modeling. The program involves 4 subsequent stages (5 sessions each): The first stage, “I feel and observe,” aims to develop body- and self-awareness. The second stage, “I discover my body potential,” aims to promote motor competence and self-regulation. The third stage, “I imagine in my body,” aims to stimulate self-regulation and emotion communication. Finally, the fourth stage, “I communicate in relationship,” focuses on relationship skills.

Along with the intervention with children, preschool teachers engage in a 25-hour training focused on the underlying principles of OUT to IN: namely, the importance of socio-emotional competencies for health, well-being and learning, the educational and developmental value of the outdoors, the potentialities of body-oriented approaches for preschoolers’ development and learning, among others. Moreover, preschool teachers also participate in 20 body-oriented biweekly 20-min sessions. Sessions involve relaxation activities, and are structured in 4 moments: activation, body awareness, body self-regulation and symbolization. Sessions are facilitated by a psychomotor therapist, with a bachelor and a master in Psychomotricity, and expertise in bodily expression and movement. The psychomotor therapist has weekly supervision with a second therapist from the research team that developed OUT to IN, with experience in psychomotor practice and supervision.

### 2.6. Data analysis

A descriptive analysis of sociodemographic and outcome variables was performed. As the Kolmogorov–Smirnov test evidenced that most variables did not have a normal distribution, intervention effects were examined through non-parametric statistics. Wilcoxon Test was used for intragroup comparisons between baseline and post-intervention. Mann–Whitney test was used to compare the results between the OUT to IN Group and the Control Group at the baseline, and the score changes from baseline to post-intervention between the two groups. Effect sizes were calculated following the guidelines of Fritz et al. (2012) for non-parametric statistics (Mann–Whitney test) and were reported as eta-squared (*η*^2^), with cut-off values of 0.01, 0.06, and 0.14 for small, medium, and large effects, respectively (Cohen, 1988). The delta value (Δ%) of proportional change between each moment (baseline, post-intervention) was calculated using the formula: Δ% = [(post-intervention – baseline)/baseline] x 100. Statistical analyses were carried out using IBM SPSS Version 27 (IBM Corp, 2017). For all statistical tests, significance was set at *p* value < 0.05. The results are expressed as mean and standard deviation, or mean and 95% Confidence Interval.

## 3. Results

As shown in Tables 2, 3, there were no statistical differences between groups at baseline, except for empathy as rated by preschool teachers, which was higher for the CG compared to the OUT to IN Group (*U* = 4407.5; *p* = 0,045).

**Table 3:** Scores (baseline and post- intervention), changes of scores, and effect sizes on relationship skills.

	Baseline (*M*, SD)		Post-intervention (10 weeks) (*M*, SD)		Difference between means *M* (%95 CI)		Value of *p*
	OUT to IN group	Control group	OUT to IN group	Control group	OUT to IN group	Control group	
Parents							
Empathy	3.91 (0.47)	3.86 (0.46)	4.02 (0.48)**	3.92 (0.40)	0.11 (−0.1.00, 1.13)	0.06 (−0.50, 1.63)	0.151
Cooperation	3.83 (0.40)	3.73 (0.46)	3.91 (0.39)*	3.83 (0.32)	0.08 (−0.50, 1.00)	−0.10 (−0.75, 2.00)	0.866
Sociability	3.71 (0.51)	3.74 (0.47)	3.83 (0.43)**	3.79 (0.47)	0.12 (−0.75, 1.38)	0.04 (−1.75, 1.50)	0.297
Communication	1.46 (0.25)	1.44 (0.21)	1.38 (0.25)**	1.36 (0.23)*	−0.08 (−0.85, 0.60)	−0.08 (−0.60, 0.45)	0.784
Preschool Teachers							
Empathy	3.94 (0.58)^a^	4.09 (0.63)	4.16 (0.60)***	3.82 (0.72)***	0.23 (−0.67, 1.67)	−0.26 (2.33, 1.00)	<0.001
Cooperation	3.89 (0.69)	3.86 (0.84)	4.00 (0.74)**	3.82 (0.69)	0.11 (−1.33, 1.67)	−0.03 (−1.00, 2.67)	0.004
Sociability	4.12 (0.54)	4.07 (0.53)	4.34 (0.52)***	3.98 (0.57)	0.22 (−0.12, 1.00)	−0.10 (−2.33, 1.00),	<0.001

^a^Between-group comparisons at baseline through Mann–Whitney Test. *Within-group comparisons through Wilcoxon Test (*p < 0.05; **p < 0.01; ***p < 0.001). *p* for 10- week changes between both groups through Mann–Whitney Test.

Several within- and between-group differences were found for self-regulation (Table 2). Significant improvements were observed after the 10-week intervention for the OUT to IN Group in Day and Night scores (49.4%, *p* < 0.001) and HTKS scores (165%, *p* < 0.001). No significant pre-post differences were found for the CG. Mann–Whitney test analysis on change scores showed significant pre-post differences between groups in the Day and Night task (*U* = 3014.5; *p* < 0.001) and the HTKS task (*U* = 1,663; *p* < 0.001), yielding a positive impact of the intervention. Large effect sizes were found for Day and Night (*η*^2^ = 0.142) and HTKS (*η*^2^ = 0.331).

Table 3 shows the results on relationship skills. Regarding within-group results, after 10 weeks children from the OUT to IN Group had higher scores on empathy (2.75%; *p* = 0.008), cooperation (2.13%, *p* = 0.019), and sociability (3.22%, *p* = 0.008), rated by parents, and on empathy (5.75%, *p* < 0.001) cooperation (2.89%, *p* = 0.003), and sociability (5.35%, *p* < 0.001), rated by teachers and had lower scores on emotion communication, as rated by parents (−5.36%, *p* = 0.002) Moreover, after the 10-week intervention period, children from the CG had lower scores on emotion communication, as rated by parents (−5.24%, *p* = 0.007), and on empathy (−6.38%, *p* < 0.001), as rated by preschool teachers.

Mann–Whitney test analysis on change scores showed significant pre-post differences between groups in empathy (*U* = 2,624; *p* < 0.001), cooperation (*U* = 4,028; *p* = 0.004), and sociability (*U* = 3478.5; *p* < 0.001) as assessed by the preschool teachers. A small effect size (*η*^2^ = 0.012) was found for empathy as rated by parents. Regarding preschool teachers’ assessments, small, medium and large effect sizes were found for cooperation (*η*^2^ = 0.036), sociability (*η*^2^ = 0.078), and empathy (*η*^2^ = 0.172), respectively.

## 4. Discussion

Body-oriented interventions and outdoor time seem to benefit preschoolers’ social–emotional development, a foundation for mental health. We investigated the effectiveness of OUT to IN, a body-oriented intervention program for preschoolers, combining physical play and relaxation activities, that was implemented in the kindergarten outdoors. The findings of the present study suggest that OUT to IN, effectively promotes preschoolers’ social–emotional competence. In particular, OUT to IN showed to increase self-regulation, empathy, cooperation, and sociability. The mostly large effect sizes of these increases suggest the effectiveness of OUT to IN in enhancing preschool children’s self-regulation and relationship skills. To the best of our knowledge, this is the first study to examine the effects of a body-oriented program implemented outdoors on preschoolers’ social–emotional competence.

OUT to IN sessions combined a sequence of exercise play and playful relaxation. Both play and relaxation, give children opportunities to feel and become aware of their bodies, either in movement or stillness (Veiga et al., 2022). Several theories have emphasized the critical role of perceiving bodily states (e.g., Damasio, 1994; Barsalou et al., 2007; Niedenthal, 2007; Fuchs and Koch, 2014) for social–emotional development. Indeed, such bodily awareness is an important component of the emotional experience and seems to facilitate self-regulation (Barrett et al., 2001; Füstös et al., 2013). Besides, both body-oriented approaches (i.e., play and relaxation) involve controlling bodies and mind, particularly by suppressing or countermanding movement and thoughts, therefore stimulating self-regulation. It is important to note that previous studies (Flook et al., 2015; Murray et al., 2018) that implemented relaxation intervention in early-childhood education settings did not show improvements in preschoolers’ self-regulation. However, these studies used sedentary/passive relaxation methods, such as progressive muscle relaxation (Murray et al., 2018) or mindfulness (Flook et al., 2015). Considering that children at such a young age should not be sedentary for extended periods (World Health Organization, 2019) and prefer intermittent type (passive/active) activity (Timmons et al., 2007), such inconsistent findings previously reported (Dias Rodrigues et al., 2022a) might suggest the importance of using physically active approaches, such as exercise play, and active relaxation when aiming to facilitate preschoolers’ self-regulation. Besides, the outdoor environment is also known as a facilitator of physical activity and self-regulation (Tillmann et al., 2018; Johnstone et al., 2022). In fact, the other known study that implemented a combined play-relaxation program (Lee et al., 2020) used more sedentary forms of play (i.e., loose parts play) and relaxation (i.e., mindfulness), that was experienced indoors.

It is important to note that, in line with other previous studies (Solomon et al., 2018; Richard et al., 2019) that also used play to improve preschoolers’ social–emotional competence, OUT to IN did not effectively decrease externalizing behaviors. While those other studies (Solomon et al., 2018; Richard et al., 2019) used role play, OUT to IN used exercise play. This more active form of play had been hypothesized by a previous systematic review (Dias Rodrigues et al., 2022a) to be important to help children learn to regulate their impulses. However, our findings did not confirm this hypothesis. Possibly, only an intervention based on rough-and-tumble play, which involves physical contact with peers, sharing the winning and losing, and more intense arousal, can help preschoolers learn to regulate their behavior. Although previous studies with older children (Carraro et al., 2014; Carraro and Gobbi, 2018) point in this direction, no study has yet examined the effects of an intervention based on rough-and-tumble play on preschoolers’ externalizing behaviors.

Nonetheless, our findings show that OUT to IN effectively improves relationship skills, such as empathy, cooperation, and sociability, which is in line with a previous systematic review that showed moderate evidence for the positive effects of play-based interventions on empathy and social interactions (Dias Rodrigues et al., 2022a). According to the authors, over and above the type of play, the social level of the intervention is the most critical component for the improvement of relationship skills. In fact, it should be emphasized that OUT to IN sessions, are carried out with the whole group of children, and involve a progression in terms of the frequency and complexity of social interactions. While the first sessions are focused on the child’s self-awareness, throughout the program there is a progressive approach to the others, implicating the empathic observation and response to the other, cooperation and problem solving.

Concerning relationship skills rated by preschool teachers, it is also important to note that while children who participated in the OUT to IN intervention increased their competencies after the 10-week period, children in the inactive control group decreased their competencies. These findings denote the importance of an intervention program for setting children on a positive trajectory for ongoing development. OUT to IN seems to protect preschoolers, uplifting them in their social relationships, which are known to be critical for their health and well-being (Goswami, 2012). The non-significant changes in relationship skills rated by parents might be related to the fact that parents do not have as many opportunities to observe their children in social contexts as preschool teachers do, limiting their appreciation of relationship skills and their sensitivity to changes in this domain (Huber et al., 2019).

Finally, our findings show that parents reported a decrease in emotion communication. Every OUT to IN session ended with a moment of symbolization when children were invited and guided to reflect on the sensations felt during exercise play and relaxation activities. Despite this specific moment to elaborate and express themselves through expressive mediators (e.g., paint, dance) such non-verbal approach might not have been enough to improve emotion communication. Moreover, although the outdoor context particularly favors body expressiveness, it poses some constraints (e.g., acoustic, intimacy) to emotional communication. The improvement of emotion communication would possibly require a calmer moment (indoors) after the session, where children could give words to their gestures, poses, and expressions.

The role of preschool teachers on the success of the program should also be acknowledged. As other studies showed (Reder et al., 2000; Justo, 2008), adults’ social–emotional competencies are critical for children’s social–emotional development. Indeed, before the beginning of the intervention, preschool teachers participated in a 25-hour training that increased their knowledge and competence regarding preschoolers’ social–emotional competence and the role of body-oriented approaches and outdoor time for social–emotional well-being. Besides, preschool teachers engaged in relaxation sessions, during the same period of children’s intervention. Such empowerment of preschool teachers’ knowledge and competence on the social–emotional domain, might have been important for the success of the intervention with children. Future studies would benefit from a planned examination of which specific components (children’s intervention, preschool teachers’ intervention, or combined interventions) of the program contribute to children’s outcomes. Moreover, future studies should also add a parent component to the intervention, as recent research indicates a stronger impact of children intervention when combined with parents’ intervention (Neville et al., 2013).

### 4.1. Implications for practice

This study’s findings reinforce the outdoors’ potential for preschoolers’ social–emotional competence. Indeed, the outdoors should deserve the same attention as the indoors, and more opportunities should be created for feeling, moving, and expressing own body outside, either by parents, preschool teachers, local communities, and policymakers.

The positive effects of OUT to IN on social–emotional competence also highlight the importance of giving preschool-aged children opportunities to experience and integrate bodily (interoceptive and proprioceptive) sensations, reflect on them, and express these corporeal experiences, verbally and non-verbally. Body-oriented approaches, such as physical play, relaxation, and dance, are a rich context for stimulating such somatic repertoire. Policymakers should acknowledge that emotions are embodied by nature, and social–emotional learning is much improved when children get the chance to identify the bodily sense of their own emotional experience. Henceforth such body-oriented practices should integrate the early-childhood education curriculum and be valued within the interaction between teachers and families. Indeed, teachers should clarify the families about the importance of these practices (e.g., physical play, relaxation, dance), especially outdoors, and support families to offer their children more bodily experiences outdoors.

Finally, one should remember that children learn to become aware and regulate their emotions, mainly by modeling, observing, and talking about emotions with knowledgeable others, such as their teachers. That is, self-aware and self-regulated teachers are critical for self-aware and self-regulated children. However, teachers face stressful conditions daily, feeling discouraged and burnt out. Considering that “teachers are the most important school-related factor impacting student learning” (OECD, 2020, p. 41), the findings of this study encourage early childhood education policymakers to provide teachers with relaxation-based intervention programs in order to develop their social–emotional competence. The development of these competencies should be focused either in pre-service education, in continuous education. These moments, where teachers can have the opportunity to feel and regulate their bodies and emotions, can help them recognize their everyday life emotions and proactively regulate how they behave and interact with children, contributing to children’s social–emotional learning.

## 5. Conclusions and limitations

OUT to IN showed to be an effective body-oriented intervention program in improving children’s self-regulation and relationship skills, which are recognized foundations for mental health and well-being. Nonetheless, it is important to note that this study has some limitations, such a lack of an active control group. Moreover, although the OUT to IN group and the control group were similar at most baseline measures, and group allocation was randomized, children were nested within classrooms. Thus, future research could use randomized control trials with random assignment at the individual participant level. Also, future studies should combine questionnaires with observational methods in order to have a more ecological assessment of social-relationship skills. Finally, further studies must include a post-intervention follow-up in their design to evaluate the long-term effectiveness of the OUT to IN intervention in self-regulation and relationship skills.

## Data availability statement

The raw data supporting the conclusions of this article will be made available by the authors, without undue reservation.

## Ethics statement

The studies involving humans were approved by University of Évora Ethics Committee. The studies were conducted in accordance with the local legislation and institutional requirements. Written informed consent for participation in this study was provided by the participants’ legal guardians/next of kin.

## Author contributions

GV and JM contributed to the design and conception of this study. GV, DG, and GDS cautiously designed the intervention program. GV and CP supervised the implementation of the study. DG collected data, which was analyzed by GV, DG, and JM. GV wrote the first draft of the manuscript. All authors contributed to manuscript revision, read, and approved the submitted version.

## Funding

This work was co-financed by the Calouste Gulbenkian Foundation’s Knowledge Academies program and by the University of Évora.

## Conflict of interest

The authors declare that the research was conducted in the absence of any commercial or financial relationships that could be construed as a potential conflict of interest.

## Publisher’s note

All claims expressed in this article are solely those of the authors and do not necessarily represent those of their affiliated organizations, or those of the publisher, the editors and the reviewers. Any product that may be evaluated in this article, or claim that may be made by its manufacturer, is not guaranteed or endorsed by the publisher.
